# Supplementary material for: Assessing receptive verb knowledge in late talkers and autistic children: advances and cautionary tales
Source: J Neurodev Disord. 2023 Dec 13;15:44. doi: 10.1186/s11689-023-09512-x (PMC10717976; doi:10.1186/s11689-023-09512-x)
Supplement: Supplementary file 1 — Additional file 1: Appendix A. List of trials for Experiment 1. [file 11689_2023_9512_MOESM1_ESM.docx]

**Appendix A: List of trials for Experiment 1.**

**Trial Number Trial** **Type** **Target Distractor**

1 N Donut Goldfish

2 V Wash Rock

3 V Tie Cut

4 N Crab Pancakes

5 V Jump Run

6 V Open Shake

7 V Clap Stretch

8 N Firetruck Bird

9 V Roll Bounce

10 V Tickle Kiss

11 V Squeeze Blow

12 N Orange Airplane

13 V Throw Kick

14 V Lick Break
