# Supplementary material for: Assessing receptive verb knowledge in late talkers and autistic children: advances and cautionary tales
Source: J Neurodev Disord. 2023 Dec 13;15:44. doi: 10.1186/s11689-023-09512-x (PMC10717976; doi:10.1186/s11689-023-09512-x)
Supplement: Supplementary file 2 — Additional file 2: Appendix B. List of trials for Experiment 2. [file 11689_2023_9512_MOESM2_ESM.docx]

**Appendix B: List of trials for Experiment 2.**

Order of trials varied between lists. The target for List 1 was the distractor for List 2 and vice versa.

**Trial** **Type** **Target (List 1) Target (List 2)**

N Cookie Banana

N Donut Goldfish

N Firetruck Bird

N Crab Pancakes

N Giraffe Rocketship

N Orange Airplane

N Grapes Squirrel

V Hug Feed

V Pour Drink

V Wash Rock

V Tie Cut

V Eat Push

V Jump Run

V Open Shake

V Read Rip

V Clap Stretch

V Roll Bounce

V Lift Pull

V Spin March

V Dance Cry

V Bite Drop

V Tickle Kiss

V Squeeze Blow

V Throw Kick

V Lick Break
